# Accuracy of CAD/CAM-fabricated bite splints: milling vs 3D printing

**DOI:** 10.1007/s00784-020-03329-x

**Published:** 2020-05-21

**Authors:** Reymus Marcel, Hickel Reinhard, Keßler Andreas

**Affiliations:** Department of Conservative Dentistry and Periodontology, University Hospital, LMU Munich, Goethestrasse 70, 80336 Munich, Germany

**Keywords:** CNC milling, Three-dimensional printing, Accuracy, Trueness, Precision, Bite splints

## Abstract

**Objectives:**

The aim of this study was to investigate the accuracy of CAD/CAM-fabricated bite splints in dependence of fabrication method (milling vs 3D printing), positioning (horizontal vs vertical), selection of material, and method of deviation measurement.

**Materials and methods:**

Bite splints were 3D-printed in either horizontal or vertical position (*n* = 10) using four different resins (Dental LT, Ortho Clear, Freeprint Splint, V-Splint). As control, ten bite splints were fabricated by CNC milling (ProArt CAD Splint). The splints were scanned and deviations between the CAD-file (trueness) and between each other within one group (precision) were measured by two different software applications and methods (cloud-to-cloud vs cloud-to-mesh). Data were analyzed using univariate analysis, Kolmogorov-Smirnov, Kruskal-Wallis, and Mann-Whitney *U* tests.

**Results:**

The highest impact on accuracy was exerted by the selection of the material (trueness: *η*_P_^2^ = 0.871, *P* < 0.001; precision: *η*_P_^2^ = 0.715, *P* < 0.001). Milled splints showed the highest trueness (*P* < 0.01) but not the highest precision at the same time. Horizontally positioned 3D-printed bite splints showed the least deviations in terms of trueness while vertical positioning resulted in the highest precision. The cloud-to-cloud method showed higher measured deviations than the other methods (*P* < 0.001–*P* = 0.002).

**Conclusion:**

Milled splints show higher trueness than 3D-printed ones, while the latter reveal higher reproducibility. The calculated deviations vary according to the measurement method used.

**Clinical relevance:**

In terms of accuracy, milled and 3D-printed bite splints seem to be of equal quality.

## Introduction

In dentistry, bite splints are used for a variety of treatments. They can be used for alleviating symptoms of temporomandibular disorders or for protecting teeth from excessive occlusal forces arising from certain patients’ habits like for example tooth grinding [1, 2]. Additionally, new occlusal situations can be tested in patients facing full mouth rehabilitation [3]. Bite splints can be manufactured by different procedures. Traditionally, vacuum thermoforming, sprinkling acrylic resin, or a combination of both is used [4–6]. With the introduction of computer-aided-design/computer-aided-manufacturing (CAD/CAM) in dentistry, a digital approach for manufacturing such devices is feasible [7]. The patient’s situation can either be digitalized with an intra-oral scanner or by means of a conventional impression which is later scanned in the dental laboratory. Based on these data, the splint is then designed digitally. This allows for a more efficient and precise workflow and enables the uncomplicated reproduction of the device if it needs replacement [8, 9].

As for the CAM process, there are two possibilities available today. The splint can either be milled out of a prefabricated blank or manufactured additively. The subtractive method entails high waste of material as well as high wear of the milling burs, especially for hard materials like ceramics. Furthermore, the production of one single splint is quite time-consuming. In the case of additively manufactured bite splints, only the support structure has to be discarded and several devices can be fabricated simultaneously [10]. Though additive manufacturing (AM) has been known since the 1980s, its application in dentistry is relatively new. The use of 3D printing for the manufacturing of bite splints was first described in 2013 [11]. Three-dimensional printing consists of different technologies and covers resin processing systems as well as ceramic or metal sintering devices [12]. For the manufacturing of bite splints, mostly stereolithography (SLA) or digital light processing (DLP) are used. They process liquid resin that is either polymerized punctually by a single-laser spot (SLA) or polymerized on a larger area with a beam (DLP) [13]. Since the object is manufactured layer by layer, it is anisotropic which means that the positioning of the object in relation to the printer’s platform during fabrication has an impact on its properties [14]. Aims of this study were to evaluate if the position of additively manufactured bite splints affects their accuracy and to investigate the accuracy of 3D-printed bite splints in comparison with milled ones.

Resins for 3D printing in dentistry often based on known formulas of existing products, like temporary materials for crowns and bridges. However, their chemical composition has to be adapted for the AM process. The resin must possess the right viscosity to easily flow between the printer’s platform and the bottom of the vat after each layer. The incorporation of fillers, pigments, and photoinitiators not only influences the materials’ mechanical properties but also affects its accuracy. If the refractive index between the resin and the incorporated substances is not well adapted, laser light is scattered which leads to reduced polymerization depth and consequently to inaccuracies of the final object. Consequently, another aim was to investigate if the choice of material affects the accuracy of additively manufactured bite splints.

Different software applications are available for investigating the accuracy of an object on a 3D scale [15–18]. These applications calculate deviations between two files by superimposing them. Using this method, two variables can be measured according to ISO standard no. 5725–1:1994: trueness and precision. In this case, trueness represents the deviation of the manufactured splint from the reference (CAD-data), while precision represents the deviation between repeatedly manufactured splints [19]. Although the different software applications work in the same manner in terms of superimposing two files and calculating the deviations between both, their results can differ. This is due to the way deviations are calculated. Hence, another aim of this study was to compare the results of two different software applications and two different measurement methods within one of these software solutions.

The null hypotheses were that the positioning and the choice of material for additively manufactured bite splints would not affect their accuracy. Furthermore, no differences of 3D-printed in comparison with milled bite splints in terms of accuracy were expected. Finally, it was assumed that the results of different measurement methods would not differ from each other.

## Materials and methods

**Fig. 1:**
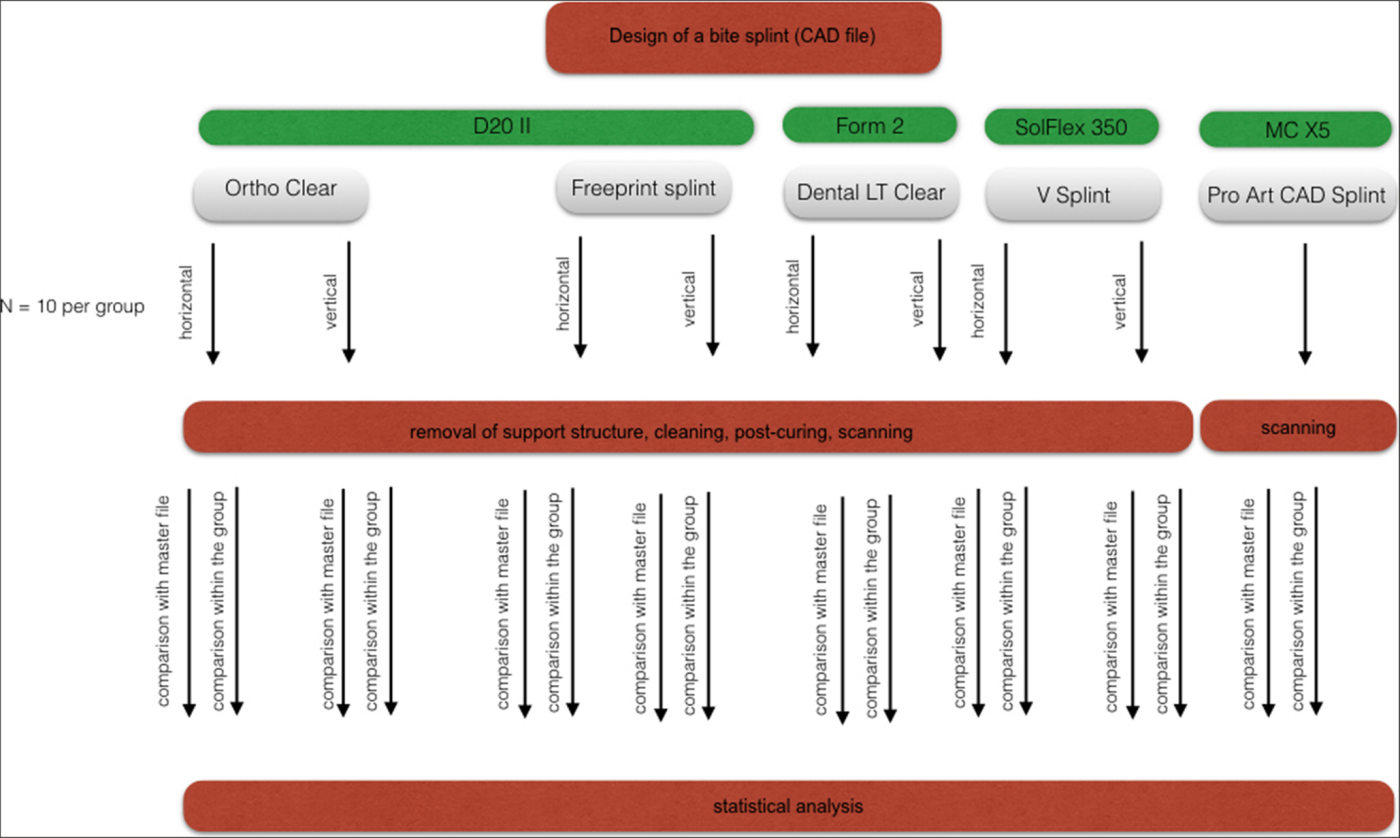
Study design

**Fig. 2:**
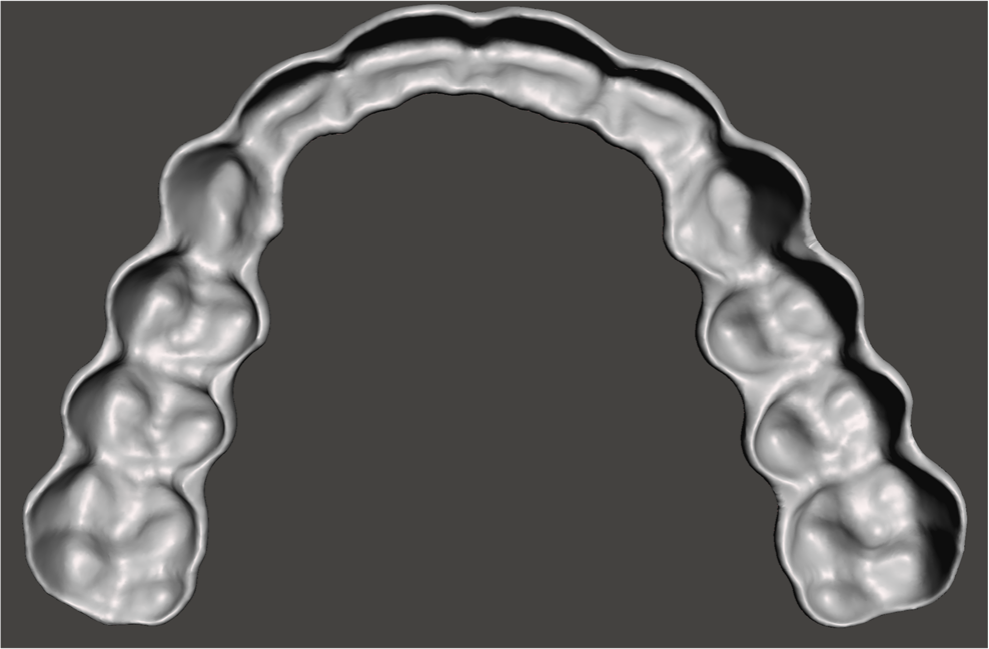
Intaglio surface of the CAD-file

The study design is presented in Fig. 1. A gypsum cast of an upper jaw was scanned (activity 885 Mark 2, Smartoptics, Bochum, Germany, working with an accuracy of 6 μm according to the ISO standard no. 12836:2015-11) and a bite splint was computer-aided-designed (exocad GmbH, Darmstadt, Germany) with a material thickness of 2 mm. The corresponding STL-file (reference) was exported (Fig. 2). Subsequently, the splint was manufactured using either an additive or a subtractive method. The investigated materials and corresponding machines are listed in Table 1. For the AM method, the splint was positioned either horizontally or vertically to the printer’s platform. Each group consisted of ten specimens (*N* = 90). The additively manufactured specimens were cleaned for 5 min in an ultrasonic-activated bath (Sonorex Super RK1022, Bandelin) of 96% ethanol (Otto Fischar GmbH) and post-cured according to the manufacturers’ specifications (Table 1). For the subtractive method, the STL-file was imported into InLab Cam 18.0 and the splints were manufactured by the 5-axis machine MC X5 with the finest bur of 0.5 mm in diameter (both Dentsply-Sirona, Bensheim, Germany). The splints’ surfaces were then scanned (activity 885 Mark 2), and the resulting STL-files were exported. Solely, the intaglio surfaces of these scans were kept by cutting off the rest of the file (Meshmixer, Autodesk, USA, version 3.5). These surfaces were compared with the reference (*n* = 90 per measurement method), representing the trueness, respectively, to each other within one group (*n* = 315 per measurement method), representing the precision. For this comparison, three different measurement methods were used: I. Cloudcompare (www.cloudcompare.com, version 2.9.2 for Mac) with the parameter *cloud-to-cloud* [Cloud1], II. Cloudcompare with *cloud-to-mesh* [Cloud2], III. GOM Inspect [GOM] (GOM, Braunschweig, Germany, version 2018) operating by default with a cloud-to-mesh measurement method. For all approaches, the STL-files were superimposed at first manually and then automatically using best-fit algorithms. To this end, the distances of each superimposition were evaluated. For this purpose, the root mean-squared (RMS) value for each superimposition was calculated as follows:

$$ \mathrm{RMS}=\frac{\sqrt{\sum {d}^2}}{\surd n} $$

where *d* is the distance between the compared cloud and the reference and *n* is the number of measurement points. The data were statistically analyzed using univariate analysis, Kolmogorov-Smirnov, Kruskal-Wallis, and Mann-Whitney *U* tests (IBM SPSS Statistics, V25, IBM Corp., Armonk, USA).

**Table 1:** Investigated materials, manufactures, machines of manufacturing, and post-curing methods

Material	Manufacturer	Machine	Layer thickness*	Post-curing
Dental LT	Formlabs (Sommerville, Massachusetts, US)	Form 2 (Formlabs) [AM]	100 μm	LC-3DPrint Box (NextDent) for 10 min
Ortho Clear	NextDent (Soesterberg, Netherlands)	D20 II (Rapidshape, Heimsheim, Germany) [AM]	50 μm	LC-3DPrint Box (NextDent) for 10 min
Freeprint Splint	Detax (Ettlingen, Germany)	D20 II (Rapidshape, Heimsheim, Germany) [AM]	100 μm	Otoflash G171 (NK Optik, Baierbrunn, Germany) for 2000 flashes
V-Splint	VOCO (Cuxhaven, Germany)	SolFlex 350 (VOCO) [AM]	50 μm	Otoflash G171 (NK Optik, Baierbrunn, Germany) for 2000 flashes
ProArt CAD Splint	Ivoclar-Vivadent (Schaan, Liechtenstein)	MC X5 (Dentsply-Sirona, Bensheim, Germany) [subtractive]	Not applicable	Not applicable

*By default, as specified by the manufactures

## Results

Descriptive statistics are summarized in Tables 2 and 3. According to the univariate analysis, the highest influence on measured distances for trueness was shown by material (*η*_P_^2^ = 0.871, *P* < 0.001), followed by position (*η*_P_^2^ = 0.664, *P* < 0.001) and measurement method (*η*_P_^2^ = 0.601, *P* < 0.001). The interaction effect of the binary and trinary combinations of the three independent parameters (material, position, and measurement method) was also significant (*η*_P_^2^ = 0.164–0.411, *P* < 0.0001). For precision, the highest influence was shown by material (*η*_P_^2^ = 0.715, *P* < 0.001), followed by measurement method (*η*_P_^2^ = 0.709, *P* < 0.001) and position (*η*_P_^2^ = 0.252, *P* < 0.001). The interaction effect of the binary and trinary combinations was also significant (*η*_P_^2^ = 0.089–0.735, *P* < 0.001). For 6 of in total 52 groups, the Kolmogorov-Smirnov indicated evidence for violation of normality assumption regarding the distribution of the data (*P* < 0.05). Therefore, the data was analyzed separately using non-parametric statistical tests.

**Table 2:** Root mean square of deviations from reference file (trueness) by additively manufactured bite splints in either horizontal or vertical building positions and one milled material (mean ± SD; min/median/max)

Measurement	Material								
	Dental LT		Ortho Clear		Freeprint Splint		V-Splint		ProArt CAD Splint
	Position								
	Horizontal	Vertical	Horizontal	Vertical	Horizontal	Vertical	Horizontal	Vertical	
Cloud1	101.2 ± 4.13; 93.8/101.3/106.9^a^	135.49 ± 7.10; 124.0/137.1/143.5^b^	93.32 ± 1.32; 90.2/93.5/94.9	99.82 ± 1.51; 97.6/100.0/101.6^a^	122.66 ± 4.67; 114.4/122.6/133.3	137.31 ± 4.65; 129.7/136.6/144.6^b^	98.92 ± 2.27.95.2/99.2/103.4^a^	108.84 ± 1.92; 106.2/108.5/112.5	67.69 ± 2.16; 65.0/67.3/71.6
Cloud2	77.39 ± 5.28; 68.9/77.3/84.9^a,A^	118.67 ± 8.00; 105.5/120.8/127.6^b^	69.43 ± 1.61; 65.5/70.0/71.1^A^	78.68 ± 2.65; 74.8/79.0/83.2^a^	104.25 ± 5.53; 95.9/104.3/116.8^A^	118.42 ± 4.18; 110.8/117.8/125.3^b^	76.05 ± 3.01; 71.0/76.4/81.8^a,A^	86.97 ± 2.39; 84.1/86.6/92.1^A^	35.08 ± 3.46; 30.0/35.0/41.1^A^
GOM	76.61 ± 4.46; 70.9/76.3/84.5^a,A^	126.82 ± 8.78; 109.1/130.3/136.8	71.30 ± 2.11; 67.7/71.5/74.2^A^	84.39 ± 5.61; 76.4/84.0/95.7^b^	101.99 ± 4.79; 92.9/102.0/107.7^A^	158.47 ± 3.52; 129.1/145.1/223.7	77.17 ± 3.22; 71.4/77.2/82.1^a,A^	87.78 ± 2.92; 82.9/88.5/91.7^b,A^	35.45 ± 2.46; 31.3/35.7/39.6^A^

^a,b^No significant differences between groups within one method

^A,B^No significant differences between groups within one material and one position

**Table 3:** Root mean square of deviation from each other within one group (precision) by additively manufactured bite splints in either horizontal or vertical building position and one milled material (mean ± SD; min/median/max)

Measurement	Material								
	Dental LT		Ortho Clear		Freeprint Splint		V-Splint		ProArt CAD Splint
	Position								
	Horizontal	Vertical	Horizontal	Vertical	Horizontal	Vertical	Horizontal	Vertical	
Cloud1	72.16 ± 3.10; 767.1/72.3/75.8^a^	68.80 ± 3.90; 64.1/67.6/72.9^b,c^	68.18 ± 2.13; 65.4/67.9/71.5^b^	60.20 ± 1.79; 58.1/60.0/63.1^d^	83.01 ± 17.27; 65.4/67.9/71.5/^e^	58.97 ± 2.69; 57.0/57.8/64.0^d^	82.73 ± 3.94; 77.3/82.3/89.3^e,A^	59.56 ± 2.43; 55.9/59.7/62.8^d^	71.01 ± 3.04; 66.4/71.2/76.4^a,c^
Cloud2	40.81 ± 5.01; 31.6/41.7/46.6^a,b^	91.62 ± 29.91; 17.6/93.4/122.2^A^	35.04 ± 3.71; 28.3/35.0/40.6^c^	22.55 ± 3.37; 19.1/21.4/28.6^d^	44.97 ± 5.66; 38.9/43.9/55.3^a,A^	19.73 ± 2.82; 15.9/19.0/25.6^d,A^	81.36 ± 2.14; 77.9/81.4/84.4^A^	21.21 ± 6.31; 77.8/81.4/84.4^d,A^	38.46 ± 6.01; 28.1/38.9/48.1^b,c^
GOM	51.80 ± 8.01; 39.6/51.5/62.4^a^	109.2 ± 13.5; 89.8/103.4/131.1^A^	31.12 ± 3.25; 26.2/30.3/37.5^b^	19.42 ± 2.62; 17.1/18.4/24.9^c^	41.22 ± 8.27; 27.2/42.9/52.0^A^	19.47 ± 2.82; 16.8/19.9/23.7^c,A^	58.22 ± 3.53; 53.4/58.2/64.7^a^	24.40 ± 2.48; 20.6/24.7/27.7^d,A^	27.38 ± 7.01; 19.2/28.2/37.2^b,d^

^a,b^No significant differences between groups within one method

^A,B^No significant differences between groups within one material and one position

Regarding trueness, within all measurement methods, the Freeprint Splint in horizontal and in vertical positions, the Dental LT in vertical position, and the V-Splint in vertical position presented the highest distances (*P* < 0.001). The overall lowest distances were recorded for ProArt CAD Splint (*P* < 0.001). Concerning the different measurement methods within one material and one position, Cloud1 presented the highest distances of all methods (*P* < 0.001). GOM illustrated higher distances than Cloud2 for the Dental LT in vertical position (*P* = 0.023), the Ortho Clear in vertical position (*P* = 0.011), and the Freeprint Splint in vertical position (*P* < 0.001). In regard to the additively manufactured specimens, horizontal positioning resulted in lower distances, regardless of the method of measurement (*P* < 0.001).

Regarding precision, within the measurement method Cloud1, the highest distances were measured for the Freeprint Splint in horizontal position and the V-Splint in horizontal position (*P* < 0.001). The lowest distances were recorded for the Ortho Clear in vertical position, the Freeprint Splint in vertical position, and the V-Splint in vertical position (*P* < 0.001). Within the measurement method Cloud2, the highest distances were measured for the Dental LT in vertical position and the V-Splint in horizontal position (*P* < 0.001). The lowest values were recorded for the Ortho Clear in vertical position and the Freeprint Splint in vertical position (*P* < 0.001). Within the measurement method GOM, the highest distances were measured for the Dental LT in vertical position (*P* < 0.001), followed by the Dental LT in horizontal position and the V-Splint in horizontal position. Concerning the different measurement methods within one material and one position, Cloud1 presented higher distances than the other methods for all groups (*P* < 0.001–*P* = 0.002) except for the Dental LT in vertical position at which Cloud2 and GOM registered higher values (*P* = 0.002, *P* < 0.001) and the V-Splint in horizontal position for Cloud2 (*P* = 0.481). Between the measurement methods Cloud2 and GOM, significant differences could be stated (*P* < 0.001–*P* = 0.023), except for the Dental LT in vertical position (*P* = 0.052), the Freeprint Splint in horizontal position (*P* = 0.579) or vertical position (*P* = 0.853), and the V-Splint in vertical position (*P* = 0.280). In regard to the additively manufactured specimens, vertical positioning resulted in lower distances, regardless of the method of measurement (*P* < 0.001).

## Discussion

All null hypotheses had to be rejected. The position and choice of material for additively manufactured bite splints influence their accuracy. Milled splints show a higher accuracy than 3D-printed ones. The results of different measurement methods differ from each other.

Concerning positioning, most interestingly, the results reveal a difference between trueness and precision. Horizontal positioning resulted in higher trueness but lower precision. This might be explained by the lower number of layers, which form one bite splint when it is manufactured in a horizontal position. In fact, for horizontal positioning, only about one quarter of layers is needed in comparison with a vertical position. Thus, the number of possible discrepancies between two subsequent layers is reduced and, consequently, the printed object is closer to the reference. In vertical positioning, on the contrary, more layers need to be connected to each other, and thus, the sum of repeated inaccuracies increases. On the other hand, when positioned horizontally, an over-curing effect due to a higher scattering of the laser beam seems to appear [20]. For a better connection between two layers, the polymerization extends the pre-set layer thickness. As a result, an over-curing of the previous layer occurs. This over-curing may reach an undesirable threshold on flat areas where multiple layers are polymerized successively, as in the case of the occlusal surface of bite splints when positioned horizontally. As a consequence, this inhomogeneous polymerization leads to uncalculated shrinkage behavior and to inhomogeneous polymerization of the resin. This results in deviations between each manufactured splint and thus a lower trueness. The over-curing effect may have great impact on transparent materials, as in the case of bite splints since here the light beam disperses more than in colored materials.

However, the Dental LT showed both, higher trueness as well as precision, when positioned horizontally. This material is the only one which is fabricated with a SLA printer. SLA and DLP differ in the way the resin is polymerized. For SLA, one single laser punctually polymerizes the layer, while for DLP, each layer is polymerized by a single beam. Considering the results of this study, it seems that the SLA printer has an inferior accuracy in the *z*-axis than the DLP printer. So, the Dental LT in vertical building direction demonstrates a greater standard deviation regarding precision than the other materials or the Dental LT in horizontal position. This result is in accordance with another study investigating the clinical fit of SLA-printed bite splints [21].

From a practical point of view, horizontal positioning results in less printing time since fewer layers are needed to manufacture the object. However, the support structure is set on the occlusal surface of the bite splint which requires more time to carefully remove it from this functionally important surface. In vertical positioning, more splints can be set on the printer’s platform and be manufactured simultaneously, which is highly efficient.

Regarding the Ortho Clear and Freeprint Splint, both being processed on the same printer, differences in accuracy could be stated. This may be explained on the one hand by the different pre-set layer thickness. As specified by the manufacturer, by default, the Ortho Clear is printed with 50-μm thickness while the Freeprint Splint is printed with 100 μm. The greater layer thickness of the Freeprint Splint leads to higher deviations from the reference, especially in the *z*-axis. This might explain why the Freeprint Splint in vertical position showed a higher RMS error regarding trueness than the other materials. On the other hand, differences in accuracy may be explained by the different composition of the respective materials. Its composition not only influences its mechanical parameters but also affects the accuracy with which one material can be manufactured. The accuracy depends greatly on the curing depth, which is described by the law of Beer-Lambert [22]. If there is a mismatch in the refractive index between additives and the resin matrix, the laser light is scattered significantly and accuracy declines consequently. Taking into account the results of this study, it seems that the material’s composition of the Ortho Clear is better adapted than that of the Freeprint Splint.

**Fig. 3:**
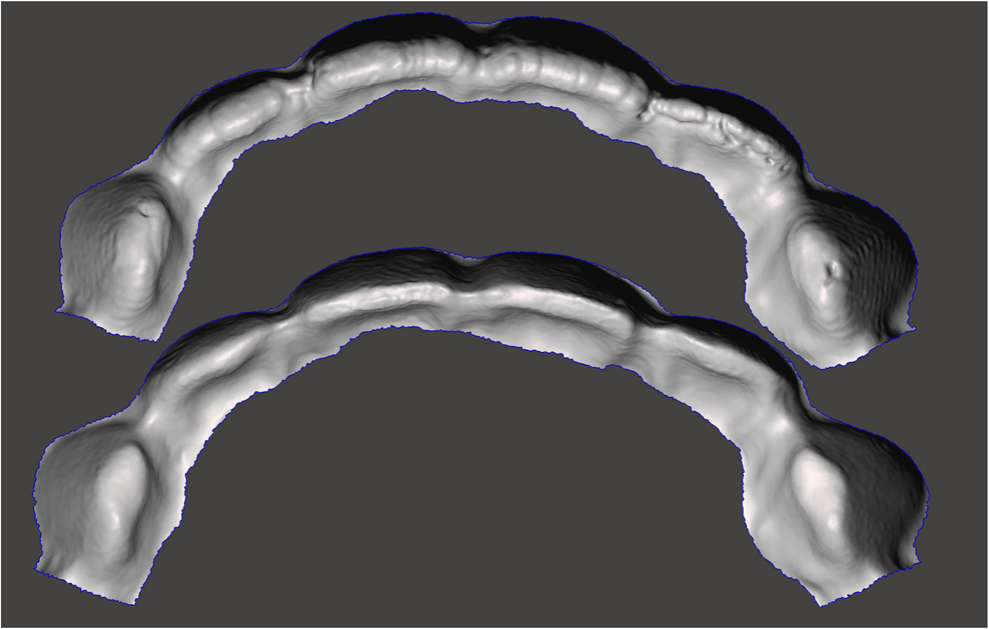
Comparison between milled (upper) and printed (lower) intaglio surface: the limitations of the burs’ diameter are obvious for the milled splints on the incisal edges

Noteworthy, milled splints showed the highest trueness but not the highest precision at the same time. From that perspective, additive manufacturing seems to offer higher reproducibility than the tested milling machine, if the objects are positioning vertically. From a technical point of view, the milling procedure differs greatly from additive manufacturing. While in 3D printing, the object is built up layer by layer; the milling machine cuts out the object from a prefabricated block. By reproducing specific geometries, the milling procedure is limited by the lowest diameter of the used burs. Concave surfaces, like the intaglio ones of the specimens, require the radius of the milling tool to be smaller than the radius to be milled. Otherwise, the milling procedure can lead to oversized reproduced geometries [23]. This restriction becomes obvious on the incisal edges of the investigated bite splints (Fig. 3.) where 3D printing shows lower deviations from the designed geometry than the milling procedure.

**Fig. 4:**
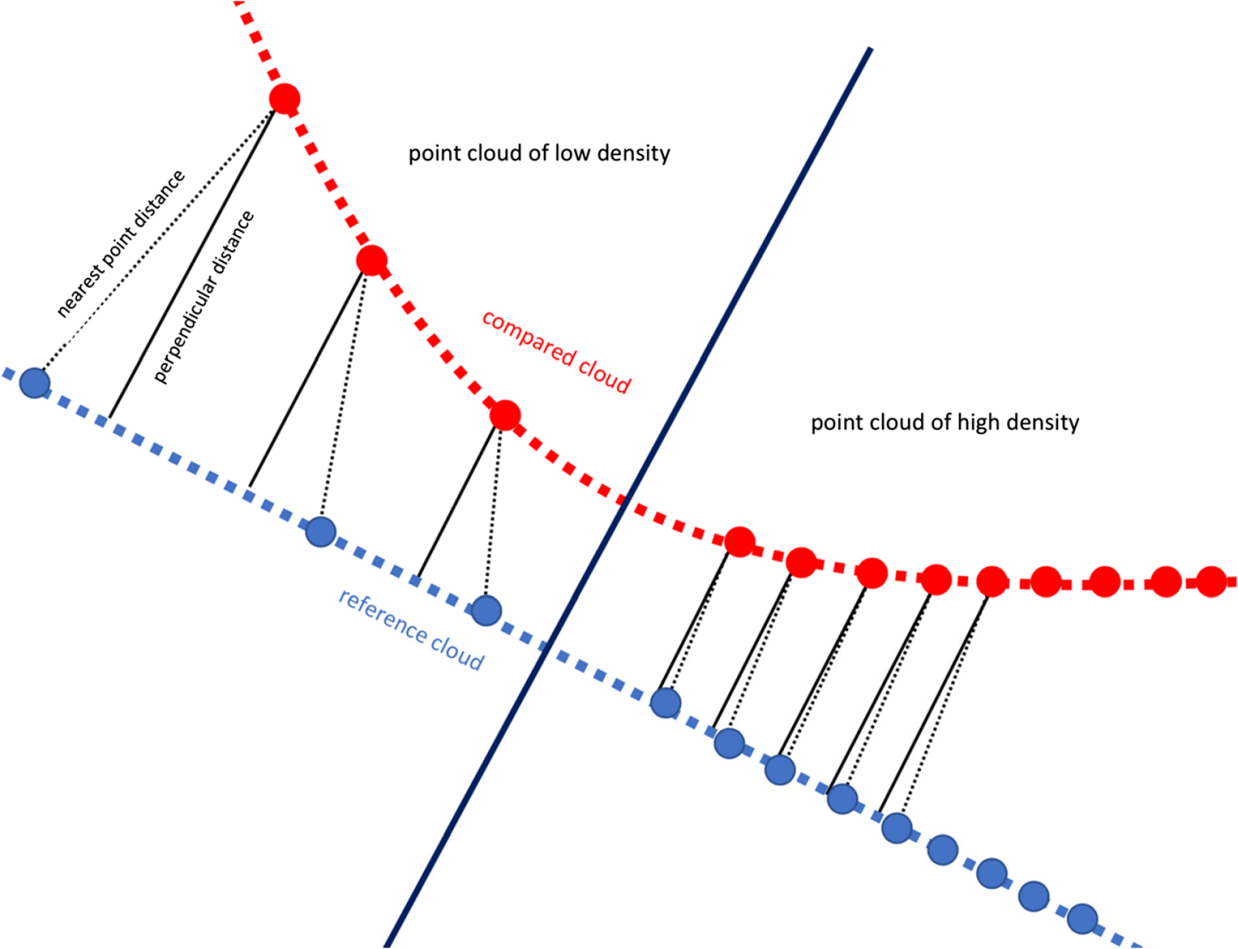
Different measurement methods: cloud-to-cloud (nearest point distance) vs cloud-to-mesh (perpendicular distance)

For assessing the deviation of two STL-files, several software applications are available. The aim of this study was to compare two of these applications (Cloudcompare vs GOM) and to compare different measurement methods within one of them (Cloudcompare: “cloud-to-cloud” vs “cloud-to-mesh”). Before measuring, the two files have to be matched. For this purpose, they are at first aligned manually and then by applying a best-fit algorithm. Subsequently, the distance from one file to the other is calculated. Therefore, two strategies can be applied: either the two STL-files are each converted to point clouds or the absolute distance between two corresponding points is measured (“cloud-to-cloud”). Alternatively, only one STL-file is converted to a point cloud and the absolute distance is defined as the way between one point and the perpendicular from this to the mesh of the STL-file (“cloud-to-mesh”) (Fig. 4). Consequently, the recorded distances may vary in dependence of the measurement method. This study stated that “cloud-to-cloud” presented higher deviations than “cloud-to-mesh.” This result can be explained by a geometrical approach. The perpendicular distance between one point and one mesh is always the shortest. However, the distance between two selected points with one of them on the mesh is greater. Both distances, the perpendicular one and the one between two selected points, may become the same if the density of the point cloud increases (Fig. 4). Nevertheless, one mesh solely represents an approach to the real object; there is always a specific secant error [24]. Based on the results of this study, it seems that the calculated deviations depend more on the measurement method than on the applied software application. For studies investigating the deviation of two or more STL-files, it therefore seems to be highly recommendable to explicitly state the used measurement method in order to compare the results with further studies.

Taking into account the results of this study, milled and 3D-printed bite splints seem to be of equal quality in terms of accuracy. While the milling procedure resulted in higher trueness, AM revealed a higher reproducibility if the splints are set in vertical position. This positioning is highly efficient if multiple splints need to be manufactured and defeats the production time of the milling procedure.

A drawback of this study is the scanner used which is not industrial “reference scanner.” However, with an accuracy of 6 μm according to the ISO standard no. 12836:2015-11, it seems suitable for the intended purpose and is in accordance with comparable studies which used a laboratory scanner as a reference as well [25–27]. Furthermore, valid comparisons can be drawn between specimens as they were all treated in the same way.

## Conclusions

Based on this study, the following conclusions can be drawn:

- Milled bite splints show higher trueness than printed ones.
- Horizontal positioning of the printed bite splints resulted in higher trueness while vertical positioning resulted in higher precision.
- In terms of accuracy, milled and 3D-printed bite splints are comparable.
- The recorded deviations depend on the measurement method.
